# Reduced Emergency Department Utilization During the Early Phase of the COVID-19 Pandemic: Viral Fear or Lockdown Effect?

**DOI:** 10.1017/dmp.2020.303

**Published:** 2020-08-12

**Authors:** Dennis G. Barten, Gideon H.P. Latten, Frits H.M. van Osch

**Affiliations:** Department of Emergency Medicine, VieCuri Medical Center, Venlo, Netherlands; Department of Emergency Medicine, Zuyderland Hospital, Heerlen & Sittard-Geleen, Netherlands; Department of Clinical Epidemiology, VieCuri Medical Center, Venlo, Netherlands

**Keywords:** COVID-19, delivery of health care, emergency preparedness, emergency service hospital, pandemics

## Abstract

**Objective:**

Since the beginning of the coronavirus disease (COVID-19) pandemic, several frontline workers have expressed their concerns about reduced emergency department (ED) utilization. We aimed to examine the changes in ED utilization during the early phase of the COVID-19 pandemic, in a country with a well-developed primary care system.

**Methods:**

A retrospective analysis of ED utilization was performed in 3 Dutch hospitals during a 60-day period, starting on February 15, 2020. The identical period in 2019 was used as a reference. ED visits were labeled as *COVID-related* (defined as COVID-19 suspected) or *non-COVID-related*. Admission rates were compared using chi-square tests, and the reduction in ED visits was assessed descriptively.

**Results:**

During the study period, daily ED volume was 18% lower compared to that of 2019. ED utilization further declined (-29%) during lockdown. Combined admission rates were higher in 2020 compared to those in 2019 (*P* < 0.001), and they were higher for COVID-19 versus non-COVID-19 ED visits (*P* < 0.001).

**Conclusions:**

ED utilization was markedly reduced during the local rise of COVID-19 in a region with a well-developed primary care system and relatively low ED self-referral rates. Although it cannot directly be concluded from the findings of our study, this observation likely reflects a complex interaction between pure lockdown effects and viral fear, which warrants further research.

The rapid worldwide spread of the novel coronavirus (severe acute respiratory syndrome coronavirus 2 [SARS-CoV-2]) caused emergency departments (EDs) around the globe to prepare for increasing coronavirus disease (COVID-19)-related patient volumes. Although it was a challenge for many EDs to simultaneously preserve sufficient capacity for “regular” emergencies, frontline workers in several disciplines and from different countries have expressed their concerns about reduced ED utilization since the beginning of the pandemic.^1-3^ Do patients avoid hospitals out of fear of contracting COVID-19, or could this phenomenon be attributed to lockdown effects, too?

In the Netherlands, the first case of COVID-19 was identified on February 27, 2020. On March 12, the Dutch Government issued social-distancing rules, including a ban on events and encouraging people to work from home. These measures were further expanded on March 23, when the government declared a modified lockdown, including school closures and stay-at-home orders for those with fever or respiratory complaints. As of June 15, there have been 48 948 confirmed cases of infection (of which 11 831 were hospitalized) and 6065 confirmed COVID-19 deaths.^4^

In this retrospective observational study, we aimed to examine the changes in ED volume during the early phase of the COVID-19 pandemic.

## METHODS

We retrospectively investigated the utilization of 3 hospital-based EDs in the southeast of the Netherlands, during a 60-day period starting on February 15, 2020. This study period corresponds with the local rise of the COVID-19 pandemic. An identical period in 2019 was used as a reference. ED 1 is a level 2 trauma center with an annual census of 25 000 patients, ED 2 is a level 2 trauma center with an annual census of 35 000 patients, and ED 3 is a level 3 trauma center with an annual census of 25 000 patients. The combined adherence area of the 3 hospitals comprises 760 000 individuals. The hospitals are situated in regions with the highest prevalence of COVID-19 in the Netherlands, with COVID-19 admission rates (ARs) ranging from 90 to 330 per 100 000 inhabitants.^4^ Before and immediately upon arrival, patients were screened for possible COVID-19 in all 3 EDs, based on their symptoms, known COVID-19 contacts, and travel history. ED visits were subsequently labeled as COVID (defined as possible or suspected COVID-19) or non-COVID-related. We retrieved these data, as well as whether or not patients were admitted to the hospital. ARs were compared using chi-square tests, and the percentage of reduction in ED visits was assessed descriptively. The study was approved by the medical ethical committee of Zuyderland Medical Center, Heerlen, the Netherlands (METCZ20200081).

## RESULTS

**FIGURE 1:**
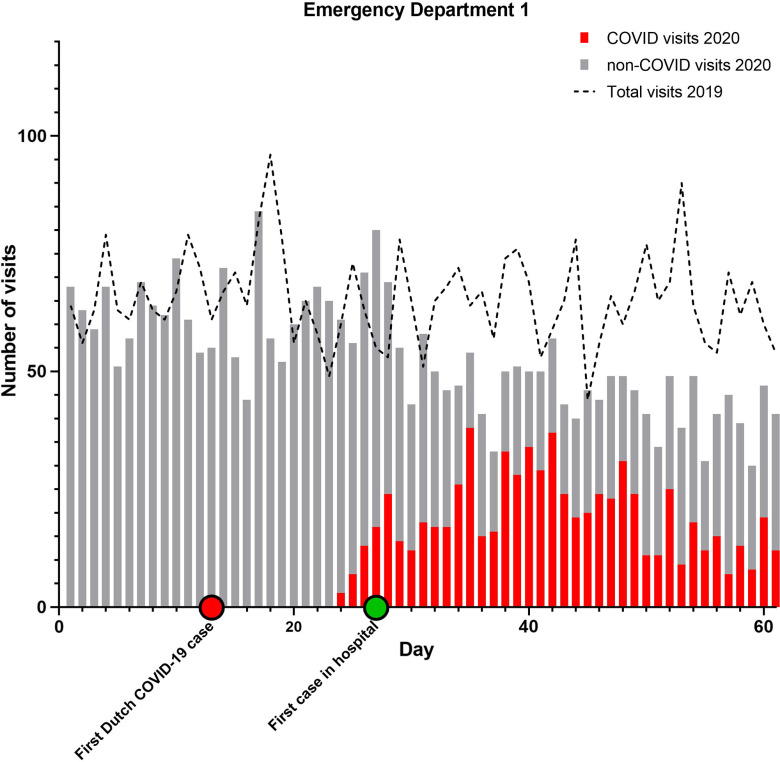
Emergency Department Utilization Pattern, Hospital 1.

**FIGURE 2:**
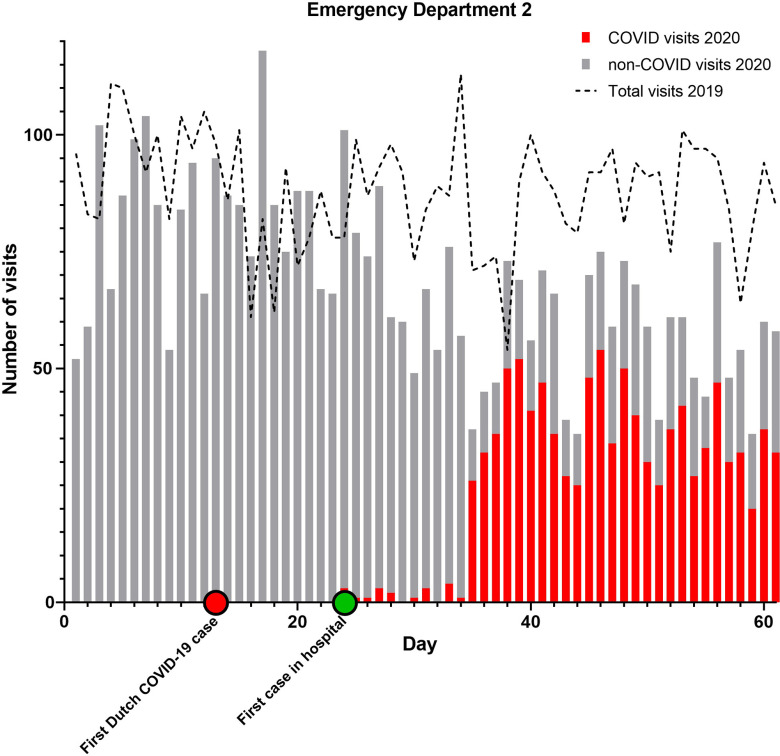
Emergency Department Utilization Pattern, Hospital 2.

**FIGURE 3:**
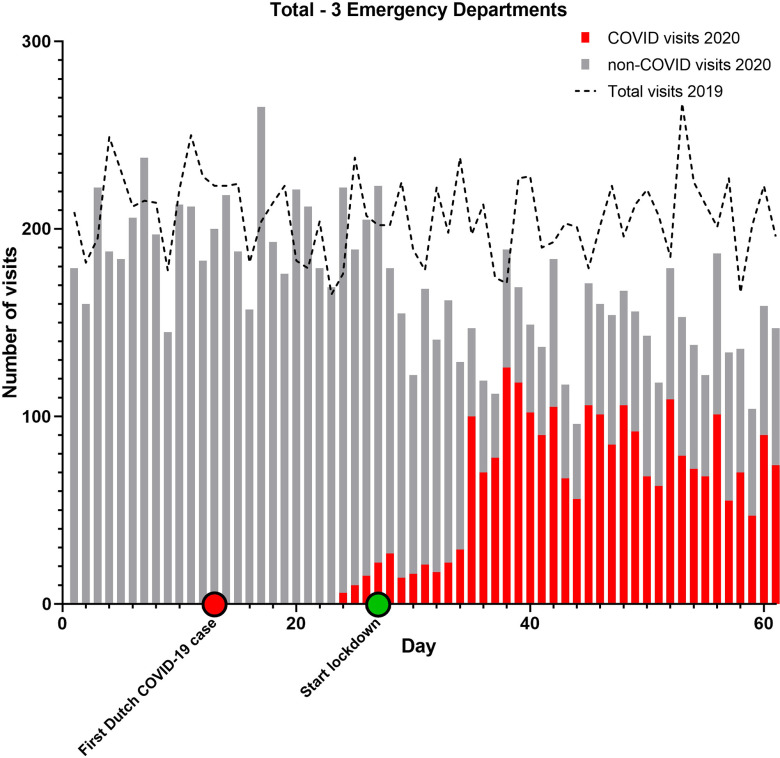
Emergency Department Utilization Pattern Hospitals Combined.

**FIGURE 4:**
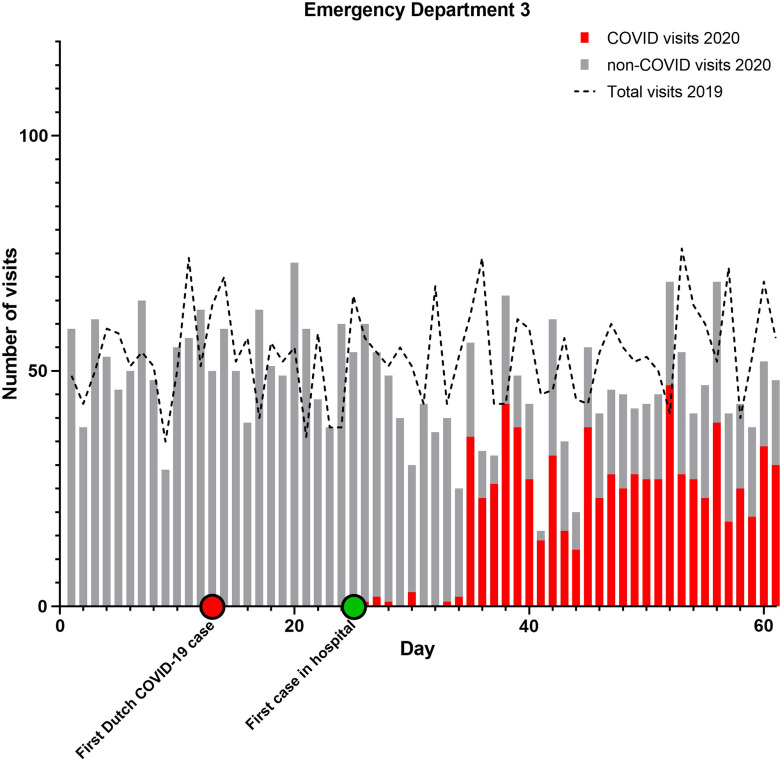
Emergency Department Utilization Pattern, Hospital 3.

From February 15 to April 16, 2020, 10 347 people visited 1 of the 3 EDs. The combined AR was 52%. Of all ED visits, 2497 (AR 67%) were COVID-related and 7814 (AR 48%) non-COVID-related (Table 1). During the corresponding 60-day period in 2019, the total number of ED attendances was 12 626 (AR 50%). The daily ED volume in 2020 was 18% lower than during the reference period (Figures 1–4).

**TABLE 1:** Emergency department utilization and admission rates

(A) ED Visits and Admission Rates, for 60 Days Starting on February 15, 2019, and the Same Date in 2020								
	2020 – Study Period							
	Total		COVID		Non-COVID		2019 – Reference Period	
	Visits	Admissions	Visits	Admissions	Visits	Admissions	Visits	Admissions
ED 1	3249	1464 (45%)	723	507 (70%)	2492	957 (38%)	3993	1528 (38%)
ED 2	4177	2362 (57%)	1009	695 (69%)	3170	1669 (53%)	5366	3167 (59%)
ED 3	2921	1577 (54%)	765	473 (62%)	2152	1104 (51%)	3267	1601 (49%)
Combined	10 347	5403 (52%)	2497	1675 (67%)	7814	3730 (48%)	12 626	6296 (50%)

(B) ED Visits and Admission Rates, for 30 Days Starting on March 16, 2019, and the Same Date in 2020 (Lockdown Period)								
	2020 – Study Period							
	Total		COVID		Non-COVID		2019 – Reference Period	
	Visits	Admissions	Visits	Admissions	Visits	Admissions	Visits	Admissions
ED 1	1389	717 (52%)	633	445 (70%)	736	272 (37%)	2002	762 (38%)
ED 2	1783	985 (55%)	998	689 (69%)	787	298 (38%)	2685	1524 (57%)
ED 3	1375	737 (54%)	756	468 (62%)	615	269 (44%)	1692	799 (47%)
Combined	4547	2439 (54%)	2387	1602 (67%)	2138	839 (39%)	6379	3085 (48%)

Combined ARs were significantly higher in 2020 compared to 2019 (*P* < 0.001 for all comparisons). Only in ED 2 the AR slightly declined. Furthermore, the ARs in 2020 for COVID-related visits were significantly higher than for non-COVID ED visits (*P* < 0.001 for all comparisons).

When comparing a 30-day period around the modified lockdown (March 16 to April 15) with the corresponding period in 2019, the decline in ED utilization is even more pronounced. ED visits were 29% lower in 2020 compared to 2019 (*P* < 0.001 for all comparisons). If the COVID-related visits in 2020 are not included in the analysis, this difference rises to 66%.

## DISCUSSION

The reduced ED utilization during the early phase of the COVID-19 pandemic has been observed in other health care systems, too, with reduction rates varying from 30 to 63%.^3,5^ Likewise, ED census decreased during the initial stages of the 2003 SARS epidemic in Hong Kong.^6^ Our study shows that ED visits also declined in a country with relatively low ED self-referral rates.^7^

The reduced ED volumes during the pandemic cannot be attributed to a single cause. Several papers have raised concerns about the impact of the fear of contracting COVID-19 in the hospital, and it has indeed been reported that patients have delayed seeking emergency care because of COVID-19 fears.^1,2^ This fear may be largely determined by the novelty of the disease and (social) media coverage.^5^ However, there probably is more than fear that keeps patients away. The (indirect) effects of lockdowns, social distancing, and improved personal hygiene have likely played a substantial role.

First, the lockdown has been associated with a vast reduction in workplace and traffic accidents.^8^ Similarly, due to the restrictions in the catering industry, we expect that nightlife-related ED attendances, such as injuries and intoxications, have declined, too. Some reports also argue that reduced access may be explained by reduced ED utilization for low-urgency complaints.^2,3^ However, this may be less evident in our EDs because of the strong gatekeeper role of primary care in the Netherlands. Self-referral rates in Dutch EDs are exceptionally low compared to other countries.^7^ Second, hospitals canceled elective surgeries and chemotherapies, which may have caused fewer ED visits for iatrogenic complications. In addition, there may have been a tendency to postpone consultations with specialists.^2^ It is likely that this tendency affected ED utilization as well. Third, there are theories that a decrease in physical exertion by patients as well as improved air quality may be associated with reduced access for pulmonary and cardiovascular emergencies.^3,9^ Finally, the lockdown and school closures were associated with a significant decrease in pediatric infectious diseases disseminated through airborne or fecal-oral transmission.^10^ Improved hand hygiene in the community may have had similar effects.

The strengths of this study are its multicenter design and the discrimination between COVID- and non-COVID-related ED visits. Similar utilization patterns were observed in all 3 EDs, underscoring the relevance of this phenomenon. Furthermore, this is the first study to determine changes in ED utilization in a country where primary care is well developed and largely responsible for relatively low self-referral rates to EDs. Its limitations are the retrospective nature of the study and the lack of adjustment for multiple potentially confounding factors. Also, the discrimination between COVID- and non-COVID-related ED visits could be debated, because triage criteria continued to evolve. However, this was probably the case in EDs all around the world. Furthermore, our observational findings do not allow for firm conclusions about the causes of reduced access for emergency conditions. In order to quantify the impact of fear and other potential variables on ED utilization, prospective, population-based research is warranted during future waves of this and other pandemics. This should include a qualitative analysis of the risk perception of patients who visited the ED and those who did not. A better understanding of underlying mechanisms may help reduce preventable injury resulting from delayed emergency care.

## CONCLUSIONS

In our cohort compromising 3 hospitals in the Netherlands, ED utilization was markedly reduced during the local rise of the COVID-19 pandemic. Although it cannot directly be concluded from the findings of our study, this observation probably reflects a complex interaction between pure lockdown effects and viral fear. To limit the potential collateral damage of fear on emergencies, emergency physicians and public health authorities should advocate the safety of hospital environments for patients with urgent medical problems.

## Abbreviations

AR: admission rate
COVID-19: coronavirus disease
ED: emergency department
SARS-CoV-2: severe acute respiratory syndrome coronavirus 2
